# Linguistic representation of vowels in speech imagery EEG

**DOI:** 10.3389/fnhum.2023.1163578

**Published:** 2023-05-18

**Authors:** Tsuneo Nitta, Junsei Horikawa, Yurie Iribe, Ryo Taguchi, Kouichi Katsurada, Shuji Shinohara, Goh Kawai

**Affiliations:** ^1^Graduate School of Engineering, Toyohashi University of Technology, Toyohashi, Japan; ^2^Graduate School of Information Science and Technology, Aichi Prefectural University, Nagakute, Japan; ^3^Graduate School of Information, Nagoya Institute of Technology, Nagoya, Japan; ^4^Faculty of Science and Technology, Tokyo University of Science, Noda, Japan; ^5^School of Science and Engineering, Tokyo Denki University, Saitama, Japan; ^6^Online Learning Support Team, Tokyo University of Foreign Studies, Tokyo, Japan

**Keywords:** EEG, speech imagery, linguistic representation, vowels, labeling syllables

## Abstract

Speech imagery recognition from electroencephalograms (EEGs) could potentially become a strong contender among non-invasive brain-computer interfaces (BCIs). In this report, first we extract language representations as the difference of line-spectra of phones by statistically analyzing many EEG signals from the Broca area. Then we extract vowels by using iterative search from hand-labeled short-syllable data. The iterative search process consists of principal component analysis (PCA) that visualizes linguistic representation of vowels through eigen-vectors φ(m), and subspace method (SM) that searches an optimum line-spectrum for redesigning φ(m). The extracted linguistic representation of Japanese vowels /i/ /e/ /a/ /o/ /u/ shows 2 distinguished spectral peaks (P1, P2) in the upper frequency range. The 5 vowels are aligned on the P1-P2 chart. A 5-vowel recognition experiment using a data set of 5 subjects and a convolutional neural network (CNN) classifier gave a mean accuracy rate of 72.6%.

## 1. Introduction

In the field of neural decoding for direct communication in brain-computer interfaces (BCIs), research is progressing for detecting spoken signals from multi-channel electrocorticograms (ECoGs) at the brain cortex (Knight and Heinze, 2008; Pasley et al., 2012; Bouchard et al., 2013; Flinker et al., 2015; Herff and Schultz, 2016; Martin et al., 2018; Anumanchipalli et al., 2019; Miller et al., 2020). If we could instead detect linguistic information from scalp EEGs, then BCIs could enjoy much wider practical applications, for instance improving the quality of life (QoL) of amyotrophic lateral sclerosis (ALS) patients, but this goal is hampered by many unsolved problems (Wang et al., 2012; Min et al., 2016; Rojas and Ramos, 2016; Yoshimura et al., 2016; Yu and Shafer, 2021; Zhao et al., 2021). While studies on spoken EEGs can leverage motor command information to help identify speech-related signals, imagined speech EEGs (that is, EEGs of silent, unspoken speech) lack that luxury (Levelt, 1993; Indefrey and Levelt, 2004), which necessitates identifying linguistic representations solely from within the EEG.

Linear predictive coding (LPC) is the widely used international standard for speech coding (Itakura and Saito, 1968; Ramirez, 2008). The LPC takes an analysis by synthesis (AbS) approach. The authors believe that EEG signal analysis would similarly benefit from linear predictive analysis (LPA) that incorporates brain wave production models (see the section “2. Materials and methods”).

Speech recognition technology was propelled by phone-labeled speech corpora such as those distributed by the Linguistic Data Consortium (LDC).^1^ Speech imagery recognition technology also needs speech corpora labeled at the phone or syllable levels. The authors used a pooling process to combine multi-electrode spectra, and manually identified and labeled chunks of discrete consonant-vowel (CV) monosyllables found in the EEG signals (see section “2. Materials and methods”).

EEG signals differ from speech signals in that unlike spoken speech, EEG signals do not exhibit coarticulation. Instead, sequences of discrete monosyllables 50 to 80 [ms] in duration are found. In the section “2. Materials and Methods,” Figure 7 shows an example of EEG spectrum of connected imagined speech, where CV are observed with no coarticulation. Coarticulation occurs at the muscular motor phase of speech production, where the movements of vocal organs effectively slur into each other.

In our vowel classification experiment involving 5 male and 1 female human subjects, we saw no marked difference of EEG signals with respect to the speaker’s sex or age. We intend to verify this in future studies by collecting more EEG data and classifying vowels. At this time, however, we attempted subject-independent recognition of the 5 vowels in Japanese language by using linguistic representations of vowels as input to the CNN.

## 2. Materials and methods

**FIGURE 1:**
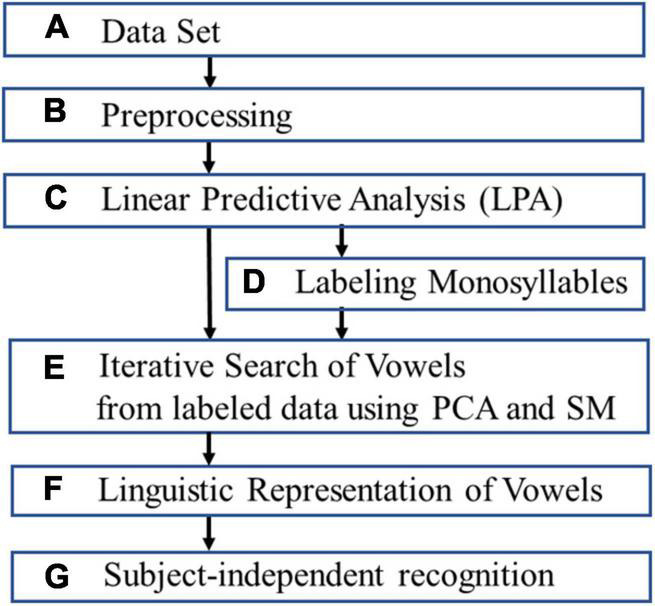
The flow chart for extracting and evaluating linguistic representation of vowels.

This section discusses extracting and evaluating linguistic representation of vowels (Figure 1).

### 2.1. Data set and protocol

**FIGURE 2:**
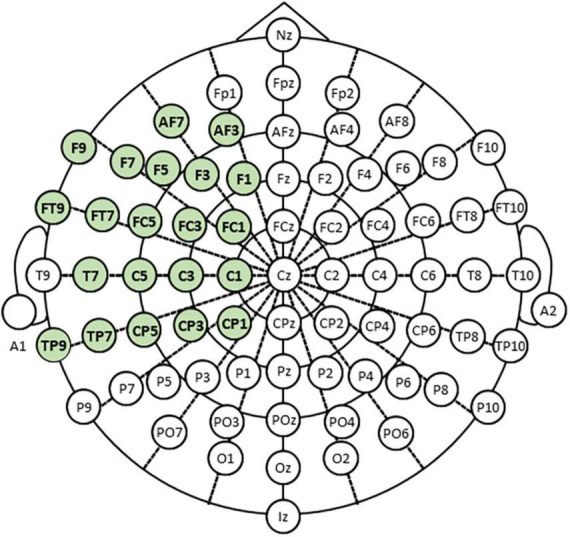
Electroencephalogram (EEG) electrode positions shown in the extended 10–20 system.

We recorded scalp EEG signals using model g.HIAMP manufactured by g.tec (g.tec medical engineering, Graz, Austria). Measurements were taken in a sound-proof and electromagnetic interference (EMI)-proof chamber at Aichi Prefectural University (APU). Figure 2 shows the placement of 21 electrodes in the extended international 10–20 system using the modified combinatorial nomenclature (MCN). The electrodes shown in green were used to measure EEG in our experiment.

The human subjects were 1 female [F1, 23 years old (y.o.)] and 4 males (M1, M2, M3, M4, 23, 22, 22, 74 y.o, respectively), all with normal hearing and right-handed. Written informed consent was obtained from all subjects prior to data collection. The experimental protocol was approved by the APU ethics committee.

Table 1 shows the imagined speech data set of 57 Japanese short syllables.

**TABLE 1:** Data set of 57 Japanese short syllables.

a	ka	sa	ta	na	ha	ma	ya	ra	wa	ga	za	kya
i	ki	shi	chi	ni	hi	mi	–	ri	–	gi	zi	–
u	ku	su	tsu	nu	hu	mu	yu	ru	–	gu	zu	kyu
e	ke	se	te	ne	he	me	–	re	–	ge	ze	–
o	ko	so	to	no	ho	mo	yo	ro	–	go	zo	kyo

**FIGURE 3:**
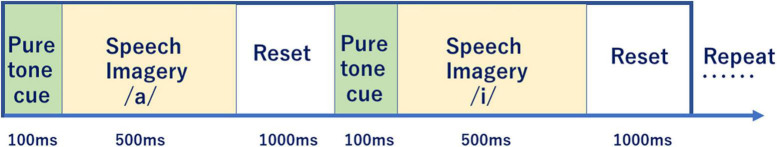
Electroencephalogram data protocol timing.

Figure 3 shows the EEG data timing protocol. Each subject imagines 57 short syllables 5 times.

### 2.2. Preprocessing of EEG data

**FIGURE 4:**
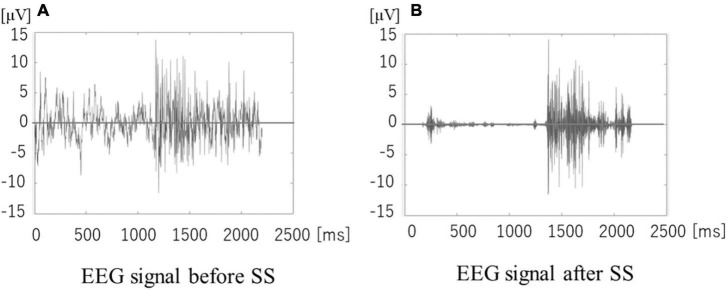
**(A,B)** Electroencephalogram before and after spectral subtraction (SS).

Electroencephalogram data was preprocessed as follows. First, we removed DC bias from the raw 21-channel EEG signal sampled at 512 [Hz], where DC bias $\bar{d\,c\,\left(n\right)}$ is the averaged value at 100 [ms] intervals, and is reduced from every sample (x(*n*) - $\bar{d\,c\,\left(n\right)}$). Second, a 128-point Fast Fourier Transform (FFT) of the 48 [ms] Hann-windowed segment is applied every 24 [ms] after zero-padding with 104 points to improve the frequency resolution. Third, noise spectrum in EEG is reduced by using a noise spectral subtraction (SS) algorithm (Boll, 1979). We obtain the mean noise spectrum $\bar{N\,\left(k\right)}$ from the initial time slot before starting of the imagined speech, which we subtract from the EEG spectrum X(k) to yield a de-noised EEG (Figure 4 shows the EEG signal of /a/ measured at TP7 before and after SS). Fourth, we apply a band-pass filter (BPF) with a pass band of 80–180 Hz on X(k), and then convert the spectrum to time waveform by applying inverse FFT (IFFT). We use the EEG spectrum of the high-γ band because the literature states that high-order cognitive functions are found in the over-γ band (Heger et al., 2015).

### 2.3. Linear predictive analysis (LPA)

**FIGURE 5:**
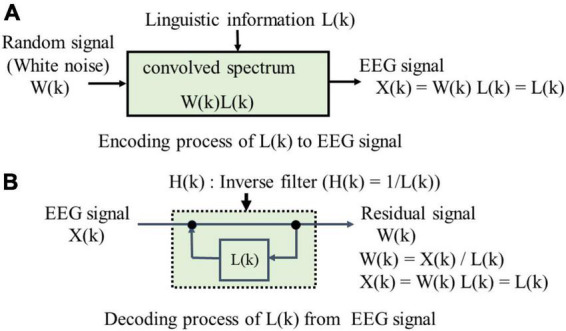
Linear predictive analysis (LPA) for EEG signal.

Figure 5 shows encoding and decoding process of linguistic information L(k) that comprise the LPA of EEG signals in which two-information sources of LPC is modified into one-information source of random signal and then L(k) is convolved. (A) in Figure 5 shows the encoding process of L(k), where the EEG spectrum X(k) is convolved with an input spectrum of random signal W(k) and the spectrum L(k) of linguistic information. Linear prediction of order p in EEG time series {x(n)} is represented by Eq. (1).


(1) $$-\hat{x}\,\left(n\right)=a_{1}\,x\,\left(n-1\right)+a_{2}\,x\,\left(n-2\right)+\ldots +a_{p}\,x\,\left(n-p\right)$$

Eq. (1) shows that the predicted value $\hat{x}\,\left(n\right)$ is represented by linear combination of {*x*_*n*−*p*_}. Here, the minus sign is for convenience of formula transformation. The squared error *e*(*n*)^2^ is then obtained by the following equation.


(2) $$e\,{\left(n\right)}^{2}={\left\{\text{x}\,\left(\text{n}\right)-\hat{x}\,\left(n\right)\right\}}^{2}$$

$$={\left\{a_{0}\,x\,\left(n\right)+a_{1}\,x\,\left(n-1\right)+\ldots +a_{p}\,x\,\left(n-p\right)\right\}}^{2},a_{0}=1$$

A set of {*a*_*p*_} is called linear predictive coefficients that is obtained from autocorrelation coefficients of the EEG time sequence {x(n)} by using Levinson-Durbin’s recursive algorithm (Ramirez, 2008). (B) in Figure 5 shows the decoding process, where the EEG spectrum X(k) is analyzed using an inverse filter H(k) with L(k) in a feedback loop. The EEG spectrum X(k), or the linguistic information spectrum L(k) of each electrode is obtained by Eq. (3):


(3) $$L\,\left(k\right)=1/ℱ\,\left\{a_{0}\,\delta \,\left(\text{n}\right)+a_{1}\,\delta \,\left(n-1\right)+\ldots +a_{8}\,\delta \,\left(n-p\right)\right\},a_{0}=1$$

where ℱ{} is a discrete Fourier transformation (DFT). Eq. (3) is called an all-pole model in LPC. LPC and LPA share an identical framework except that LPA’s sole information source is random noise. We analyze imagined-speech EEGs using LPA by positing an encoding process where linguistic information is convoluted and a decoding process where linguistic information is extracted using an inverse filter. The LPA spectrum L(k) is calculated by Eq. (4) after 0-padding {a_*p*_} to arrange the frequency resolution of EEG spectrum.


(4) L⁢(k)=X⁢(k)

$$=1/ℱ\,\left\{1,a_{1},a_{2},\ldots ,a_{8},0,0,\ldots ,0\right\}$$

=1/{ReX⁢(k)-jImX⁢(k)}

={ReX⁢(k)+jImX⁢(k)}/{Re2⁢X⁢(k)+Im2⁢X⁢(k)}

**FIGURE 6:**
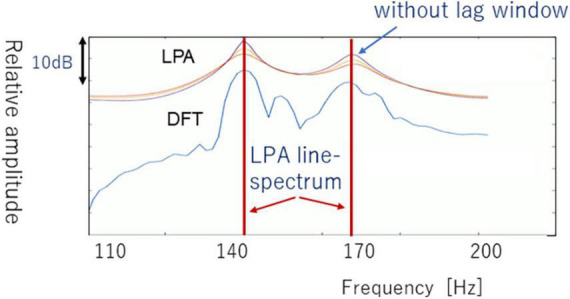
Linear predictive analysis spectrum of a vowel [a] in comparison with DFT spectrum.

Figure 6 compares an example of an LPA spectrum versus DFT spectrum. Figure 6 shows three types of LPA spectra that have different lag windows in autocorrelation domain. In this section, we do not use the lag-window, because the LPA spectrum with sharp peak is adequate for converting LPA spectrum to line-spectrum. The LPA spectrum patterns are lastly converted to LPA line-spectrum patterns by using local maximum values and inflection point that are derived from first derivative Δ(k) and second derivative ΔΔ(k); see LPA line-spectra in Figure 6.

### 2.4. Labeling monosyllables

In the case of spoken speech (that is, phones or phrases said aloud) observers can synchronize the audio and EEG signals to label speech. In the case of imagined speech however, because there is no reference time signal corresponding to the exact moment the speech was imagined (that is, spoken silently in the human subject’s mind), we need to discover how and where phones or phrases are represented in the multi-channel EEG signal. After analyzing many EEG line-spectra of phones, words, and sentences, we learned that when we integrate (or pool) multi-channel data, chunks of discrete open syllables (that is, consonant-vowel combinations, or CV) having durations of 7–9 frames (56–72 [ms]) become apparent.

**FIGURE 7:**
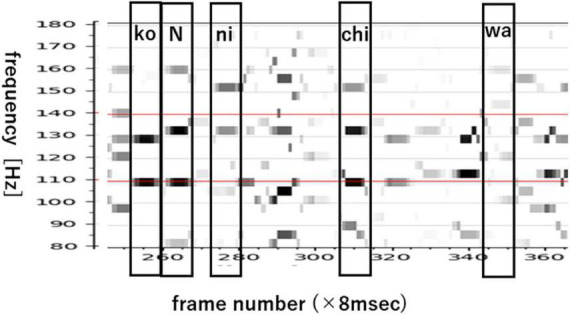
Monosyllable labels of phrase /koNnichiwa/ (good afternoon).

Figure 7 shows an EEG line-spectrum sequence that was integrated from 21 electrodes by pooling line spectra. The human subject imagined the Japanese sentence /koNnichiwa/ (”good afternoon”). Because vowels remain stable across multiple frames, CV line spectra resemble V line spectra after pooling. Also noteworthy is the fact that numerous pseudo- (or false or quasi-) short syllables appear in imagined sentences. These pseudo-short syllables seem to arise from sentence-initial /koN/ (N: the Japanese moraic nasal); /ko/ (appearing in frames 282, 320, 332,340, 360), and /N/ (appearing in frames 293, 355). When CV are imagined, many pseudo-short syllables appear alongside true (or real or genuine) speech imagery within the interval of imagined signal. In the next section, we show how we search for vowels from line-spectra data of 21-electrodes with 9 frames.

### 2.5. Iterative search of vowels from labeled data using PCA and SM

**FIGURE 8:**
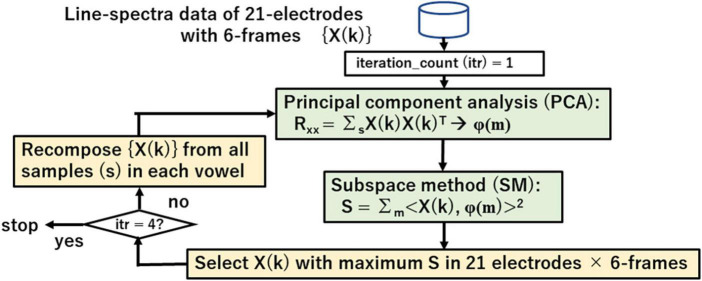
Iterative search for vowel spectra by using principal component analysis (PCA) and subspace method (SM).

Figure 8 shows the iterative search process for vowel spectra {X(k)} using principle component analysis (PCA) that visualizes linguistic information through eigen-vectors φ(m) and subspace method (SM) that searches the appropriate spectra of vowel for recomposing {X(k)} and redesigning the eigen-vector set. Eq. (5) shows the similarity between a vector X and eigen-vector φ(m) in SM.


(5) $$S=\sum_{m=1}^{M}\frac{<X,\varphi \left(m\right){>}^{2}}{{||X\,\left(k\right)||}^{2}\,{||\varphi \,\left(m\right)||}^{2}},M=8$$

Search range is fixed to the last 6 of 9 frames. The iterative search proceeds as follows:

1. Design initial eigen-vectors φ(m) of each vowel from all 21 electrodes and 6 frames.
2. Calculate similarity S between φ(m) and spectra of 21 electrodes and 6 frames.
3. Select spectrum X(k) with maximum S.
4. Recompose {X(k)} from all samples and redesign an eigen-vector set by PCA in each vowel.
5. Repeat steps 2, 3, 4 for 4 iterations.
6. Repeat all steps for all vowels.

Lastly these steps give an eigen space ψ(v, m); v = i, e, a, o, u; m = 1, 2,…, M that represents vowel v.

## 3. Results

### 3.1. Linguistic representation of vowels

The resultant eigen space ψ(v, m) likely contains the linguistic representation of vowels. The referencing vector of vowel v is given as Eq. (6).


(6) $$G\,\left(v\right)={\left[\sum_{m=1}^{M}\frac{\lambda \,\left(m\right)}{\lambda \,\left(1\right)}\,\psi \,{\left(v,m\right)}^{2}\right]}^{1/2}$$

G(v) is the accumulated spectrum with the weight λ(m)/λ(1). The magnitude of eigen-value λ(m) represents the degree of contribution to G(v).

**FIGURE 9:**
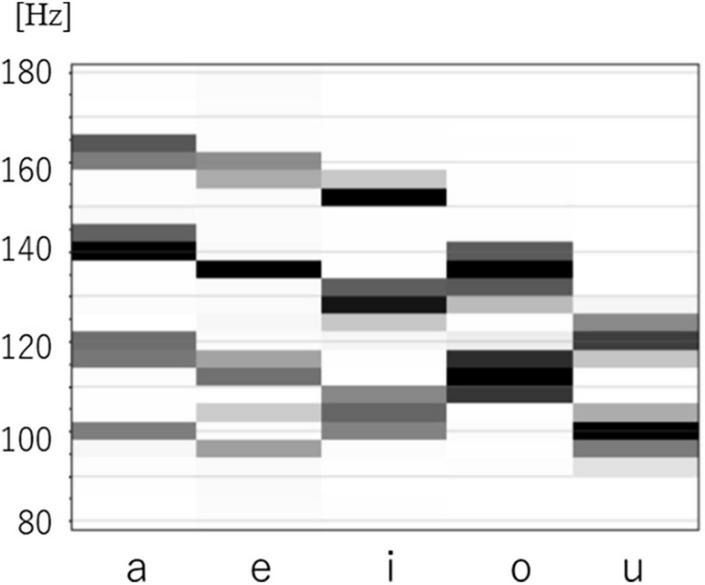
Reference vectors of five vowels.

Figure 9 shows G(v) for 5 vowels /i/ /e/ /a/ /o/ /u/. The 2 spectral peaks (P1, P2) in the upper frequency range remind us of the 2 formant frequencies (F1, F2) in audio spectra of spoken vowels.

**FIGURE 10:**
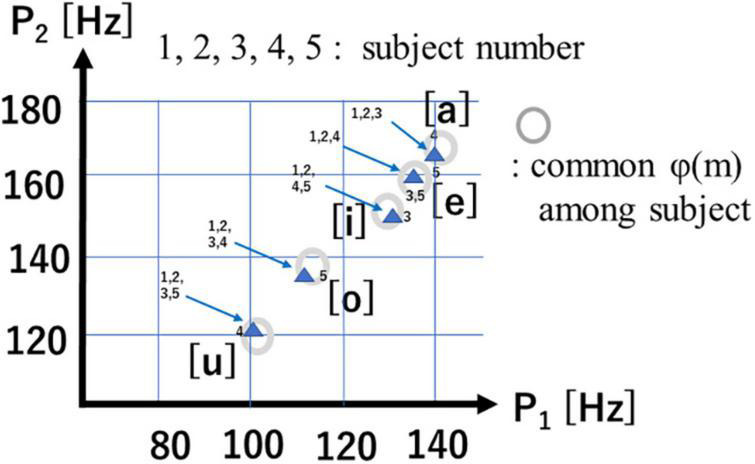
P1-P2 chart of five vowels.

Figure 10 is a scatter plot of P1-P2 values for each of the 5 vowels, with data points from human subjects (4 male, 1 female) and their mean values (Δf = 3.9Hz). Of note is the fact that the 5 vowels in the P1-P2 scatter plot roughly form a line, while cardinal vowels in a F1-F2 plot for spoken speech form a quadrilateral. Also of note is that male and female data points overlap in the P1-P2 scatterplot, while they differ in the spoken vowel F1-F2 plot (Kasuya, 1968).

### 3.2. Subject-independent recognition

**FIGURE 11:**
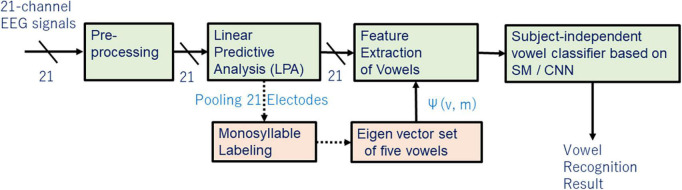
Block structure diagram of subject-independent vowel recognition system prototype.

Figure 11 shows a block structure diagram of a subject-independent vowel recognition system prototype that was built to evaluate subject-independent recognition of imagined speech vowels. The vowel classifier compares recognition results of SM and CNN. The CNN is composed of 2-dimensional CNN layers, subsampling layers (2-dimensional pooling), and fully connected layers (multi-layer perceptron or MLP).

**FIGURE 12:**
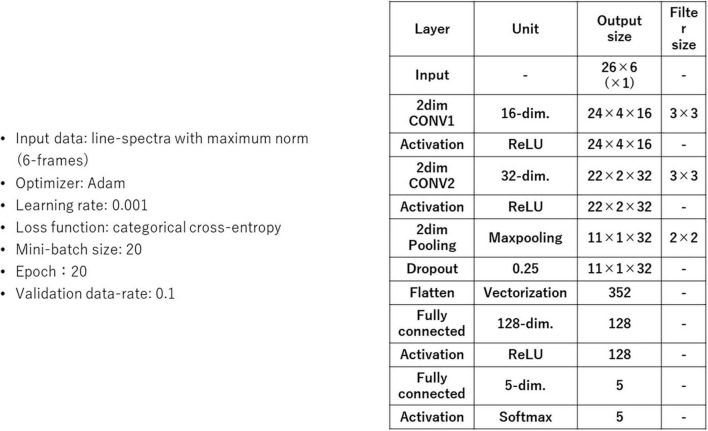
Convolutional neural network (CNN) parameters.

Figure 12 shows CNN parameters. Recognition accuracies of SM and CNN were measured by using an imagined speech corpora of 5 human subjects. Each human subject imagined the speech of /i/ /e/ /a/ /o/ /u/ 50, 50, 65, 60, 60 times respectively, for a total of 285 samples per human subject, yielding 285 × 5 = 1425 samples in the entire data set. These vowels were taken from the 57 CV in Table 1.

We trained and tested using a so-called jack-knife technique, where 4 of the 5 human subjects were used as training data, the remaining 1 human subject was used as the test data, and we repeated training and testing by alternating training and test data for all human subjects, resulting in cross-validation across 5 human subjects (that is, 1425 × 4 = 5700 samples for training, and 1425 × 1 = 1425 samples for testing). Table 2 shows results of 2 recognition experiments for imagined vowels.

**TABLE 2:** Recognition accuracies of imagined speech vowels.

		Human subjects and recognition accuracies [%]					Descriptive statistics	
		**Male 1**	**Male 2**	**Male 3**	**Male 4**	**Female 1**	**Mean**	**Standard deviation**
Classifier	Subspace method (SM)	63.5	64.2	68.4	52.6	63.5	62.8	5.25
	Convolutional neural network (CNN)	73.4	72.3	76.1	64.6	70.9	72.6	3.83

## 4. Discussion

Until now, measurements of linguistic activity in the brain have been limited to where information, that is, location measured by using PET or fMRI for instance. By contrast, what information, that is, how linguistic information is being realized, has been largely neglected. This paper described the following:

1. Hand-labeled short syllable data is extracted from the LPA line-spectra of scalp EEG signals after a pooling process.
2. Iterative search processes of PCA and SM derive eigen-vector sets for 5 vowels.
3. The reference vector G(v) of each vowel calculated from an eigen-vector set φ(m) of line spectra probably contains vowel-specific information.
4. Two prominent spectral peaks (P1, P2) are observed in the upper frequency range, and the 5 vowels are aligned on the P1-P2 chart.
5. The P1-P2 chart suggests that there are no differences in speech imagery between male and females, which would be consistent with the lack of sex differences in EEG signals.
6. A CNN-based classifier obtained a mean recognition accuracy of 72.6% for imagined speech vowels collected from 4 male and 1 female human subjects (however, Male 4 had lower accuracy).

Lopez-Bernal et al. (2022) recently reviewed studies of decoding the EEG of imagined 5 vowels. Recognition results are divided curiously into 2 groups: (1) poor performance below 40% (Cooney et al., 2020), and (2) better performance exceeding 70% (Matsumoto and Hori, 2014). Techniques that do not use labeled EEG data have no choice but to use the whole time duration (typically 1 to 2 [s]) of imagined speech to train the recognizer. Because numerous pseudo-short syllables appear alongside imagined speech, the better-performing recognizers, particularly for vowel recognition, benefit from an abundance of the same short syllables containing the vowel to be recognized. By contrast, when sentences are imagined, only the short syllable at the beginning of the sentence is abundant, and because it differs from other short syllables within the sentence, recognition accuracy may deteriorate.

Our next steps for discovering the linguistic representation in EEGs are (a) extract consonant information, (b) improve recognition accuracy of vowels and consonants, partly by increasing the imagined speech corpora, and (c) build decoding modules for isolated words and/or connected phrases for the purpose of BCI applications.

Incidentally, we are fascinated that EEG line spectra and atomic line spectra closely resemble each other.

## Data availability statement

The original contributions presented in this study are included in the article/supplementary material, further inquiries can be directed to the corresponding author.

## Ethics statement

The studies involving human participants were reviewed and approved by YI, Aichi Prefectural University. The patients/participants provided their written informed consent to participate in this study.

## Author contributions

TN, GK, and JH conceived the presented idea. YI, JH, and TN collected the EEG data. TN and YI carried out the data processing and analysis. RT and TN developed a labeling tool and labeled monosyllables on EEG data. KK and SS programed and evaluated the classification of vowel using DNN. TN wrote the manuscript with support from GK. All authors contributed to the article and approved the submitted version.

## Footnotes

1
https://www.ldc.upenn.edu
